# Cohort profile: The Halmstad University Register on Pupils with Intellectual Disability

**DOI:** 10.1002/jcv2.70028

**Published:** 2025-08-01

**Authors:** Eva Jönsson, Carin Staland‐Nyman, Paul Lichtenstein, Magnus Tideman

**Affiliations:** ^1^ School of Health and Welfare Halmstad University Halmstad Sweden; ^2^ Department of Clinical Neuroscience Karolinska Institutet Stockholm Sweden; ^3^ Department of Medical Epidemiology and Biostatistics Karolinska Institutet Stockholm Sweden; ^4^ School of Allied Health Human Services and Sport La Trobe University Melbourne Victoria Australia

**Keywords:** cohort profile, intellectual disability, register, special school

## Abstract

**Background:**

Knowledge about the living conditions among people with intellectual disabilities (ID) is globally scarce. Even in countries with good access to registers, this is often partly due to the absence of a single, comprehensive, nationwide register of individuals with ID or the inability to identify all individuals with ID within existing registers. In Sweden, the Halmstad University Register on Pupils with ID (HURPID) was established to fill this gap. HURPID is based on the population that left the Swedish Upper Secondary School for pupils with ID (USSID) in 2001–2020. Most young people with ID in Sweden are expected to attend USSID. The aims of this paper were to describe the construction, structure, and content of HURPID, to validate the register, and to provide examples of what it has been used for.

**Methods:**

Based on school leaving certificates or corresponding, personal identity number, gender, educational programme, municipality where the school was located, and graduation year were registered. HURPID has been linked with various registers in projects addressing research questions about the target group's living conditions.

**Results:**

HURPID consists of 26,910 individuals (11,078 women, 15,832 men), born in 1973–2003. Through linkages, knowledge has been gained about occupational patterns, psychiatric comorbidity, mortality, substance use, criminality and victimisation.

**Conclusions:**

HURPID demonstrates the highest completeness of individuals with ID of working age in Sweden. This makes it possible to follow the development of adolescents with ID over time. As one of the most extensive and comprehensive registers of individuals with ID in the world, it also forms a unique register internationally.

**Key points**

**What's known**

- Even countries with good access to registers often lack the ability to accurately identify all their citizens with intellectual disabilities (ID). The Halmstad University Register on Pupils with ID (HURPID) was established to fill this gap in Sweden.

**What's new**

- HURPID has been updated and expanded. It contains data on 26,910 individuals who left the Upper Secondary School for pupils with Intellectual Disability between 2001 and 2020. This paper validates HURPIDs potential as a nationwide register of adults with ID.

**What's relevant**

- HURPID makes it possible to follow the living conditions of adolescents with ID as other citizens.
- Linkages with other registers can advance scientific knowledge, influence policy, clinical and educational practices, and service development.

## INTRODUCTION

Intellectual disability (ID) is a neurodevelopmental disorder, characterised by deficits in intellectual and adaptive functioning, with severity levels ranging from mild to profound (American Psychiatric Association [APA], 2022). The global prevalence of ID is about 1% (APA, 2022; Maulik et al., 2011; McKenzie et al., 2016). Up to 85% of these have a mild severity level (Boat & Wu, 2015; King et al., 2017). Research on occupational patterns, health, and living conditions of individuals with ID is globally scarce. Even in countries with good access to registers, this is partly due to the lack of a complete, nationwide register of individuals with ID, or the inability to identify all individuals with ID within existing registers. Accurate identification of the entire population of individuals with ID allows for comprehensive longitudinal analyses which can enhance clinical visibility and help ensure provision of appropriate services. In Sweden, ID is diagnosed within various healthcare settings. Many of these are not authorised to report diagnoses to the National Patient Register (NPR) (National Board of Health and Welfare (Sweden), 2018, November 9), making this register incomplete regarding ID. Through the National Register of Municipal Support and Service for Persons with Certain Functional Impairment (LSS) (National Board of Health and Welfare (Sweden), 2022, October 20), individuals granted services due to ID or autism spectrum disorder (ASD) (Support and Service for Person with Certain Functional Impairments Act [LSS], 1993), can be identified although not distinguished from one another. However, as not all individuals with ID apply for these services, this register also remains incomplete. Similarly, the Register of students in basic and upper secondary education for adults with intellectual disability (National Agency for Education (Sweden) [NAE], 2022) is incomplete, as not all individuals with ID attend this form of education.

Although voluntary, most individuals with ID in Sweden are expected to attend the Swedish Upper Secondary School for pupils with Intellectual Disability (USSID). Thus, identifying attendees could make it possible to create a nationwide register of individuals with ID. Swedish authorities could not collect individual‐specific information about these pupils until autumn 2019 (Regulations amending the National Agency for Education's Regulations (SKOLFS, 2011:142) on data collection from organisers within the school system, etc., 2019), but Halmstad University received ethical approvals to obtain school leaving documents to establish such a register (Halmstad University Register on Pupils with Intellectual Disability (HURPID)) in 2011. USSIDs target group includes pupils assessed as not having the prerequisites to fulfil the minimum grade criteria for the mainstream upper secondary school in Sweden, due to ID or an acquired brain injury, causing a significant and permanent intellectual impairment (Swedish Education Act, 2010). Until the introduction of the latest Swedish Education Act (2010) in July 2011, it also included pupils with ASD. From 2011, new enrolments of pupils with ASD without co‐occurring ID were prohibited, but those already enroled were allowed to finish (Swedish Education Act, 2010). In practice, this means that these pupils completed their fourth and final year by 2014 at the latest, unless any study breaks occurred. HURPID data does not specify diagnoses. However, individuals with an acquired brain injury or ASD without co‐occurring ID are expected to constitute a minor proportion. The establishment of HURPID made it possible to follow the development of adolescents with ID from school age to working age in the same way as other citizens. Since then, the register has been updated and expanded with new cohorts. This article reports these changes and validates HURPIDs potential as a nationwide register of adults with ID in Sweden.

### Aims

The aims of this paper were to describe the construction, structure, and content of HURPID (graduation years 2001–2020), to validate its potential to serve as a nationwide register of adults with ID in Sweden, and to provide examples of the knowledge contributed so far by various projects involving HURPID.

## MATERIALS AND METHODS

To identify the individuals who attended the fourth year of USSID during the graduation years 2001–2020, two periods of data collection for school leaving documents were conducted. The first included the graduating classes of 2001–2011, the second, the graduating classes of 2012–2020.

All pupils who finish USSID, regardless of programme and performance, should receive a formal document (Ordinance concerning USSID, 1994; Upper secondary school ordinance, 2010), issued by each individual school (Swedish Education Act, 2010). In case of interruption from a graded programme, the grades achieved so far should be documented (Ordinance concerning USSID, 1994; Upper secondary school ordinance, 2010). Documents received or drawn up by an authority are public (Freedom of the Press Act, 1949). Consequently, school leaving documents issued by independent schools are not public. However, since 2011, they must be handed over to the municipality where the school is located (Swedish Education Act, 2010) and thus become public. Thus, since then, it should be possible to request and obtain documentation for all pupils who have left USSID, except for those who interrupted their studies from a non‐grade‐based programme or from an independent school, or for those whose school leaving documents have been classified as confidential.

### Eligibility criteria

The only inclusion criterion applied during the first data collection period was a unique personal identity number (PIN). To the extent that they could be identified, individuals who interrupted their studies before reaching year four, and individuals who appeared to have been enroled in USSID, but only had obtained grades from courses given in mainstream upper secondary school, were not included in HURPID during the second data collection period.

### Setting

The municipality in which the pupil is registered decides regarding admission to USSID. The organiser of a school unit can either be a municipality (most common), a region, or an independent school (Swedish Education Act, 2010). In September 2011, a request for school leaving documents of the individuals who left USSID in 2001–2011 was sent to Sweden's 290 municipalities via mail, asking them to return the documents either in an attached reply envelope or by e‐mail. When misconceptions about the submitted material were discovered, the municipalities were contacted again. After up to three reminders, all municipalities had responded, either by sending documents or by announcing that they had not run USSID during the requested period. Data, at least including a PIN, were obtained in February 2012 (Arvidsson, 2016) from 172 out of 198 municipalities (coverage 87%) where USSID, regardless of organiser, had been run during this 11‐year period (unpublished data made to order). Late submissions from two additional municipalities were added in 2022.

The second data collection period, covering the graduation years 2012–2020, was conducted in a similar manner. A request for school leaving documents of the individuals who left USSID in 2012–2019 was sent by e‐mail to all municipalities in Sweden in September 2020, and, in April and May 2021, to the regions that had run USSIDs during the same period (NAE 1992–). The municipalities and regions were reminded until responses were received in May 2022 from all who had reported having pupils enroled in USSID during the specified period (unpublished data made to order; NAE 1992–).

The independent school units who had pupils enroled in USSIDs fourth year during the specified period (unpublished data made to order) were approached and asked to submit their documents if they were not available in the municipal archives. Some of these school units were no longer in operation and contact was therefore not feasible.

Data that could be included in HURPID were obtained in June 2022 from 179 of the 188 municipalities (coverage 95%) where USSID (regardless of organiser) had been run during these eight years, according to the NAE (unpublished data made to order). Insofar as time allowed, follow‐up questions were directed to the school organisers, aiming to interpret the material and/or potentially gather further information.

Data for the graduating classes of 2020, covering 140 municipalities where the fourth year of USSID had been run (regardless of organiser), was obtained from the NAE (unpublished data made to order).

HURPID will be supplemented with additional cohorts every 5^th^ year. The register is continuously updated when errors are discovered. Table 1 shows the variables included in HURPID.

**TABLE 1:** Description of the variables included in HURPID.

Variable	Description	Timeliness
PIN	The minimum unit of information required for inclusion in HURPID	Applies to everyone
Legal gender	Based on the penultimate digit of the PIN	As above
Date of birth (YYYYMMDD)	Based on the eight first digits in the PIN	As above
Graduation year	Based on the date when the document was issued, the date of the most recently completed course, or on other information from the issuer	Applies to everyone, except for three individuals who left USSID in unspecified years between 2012 and 2019
Municipality	The municipality where the pupil received her or his education (regardless of school organiser)	Applies to everyone, except for those who were instead either registered under a region that ran schools in more than one municipality between 2001 and 2011 or under an upper secondary school association that ran schools in more than one municipality in 2007 (total *n* = 86)
Municipal/Upper secondary school association	Municipalities that jointly organise at least one USSID unit	Applies to the graduating classes of 2001–2011
Region	Regional organiser	As above
Educational programme	Orientation	
Individual	General life skills training	Applies to the graduation classes up to and including the classes of 2016^a^
	Vocational training	As above
	No orientation	Applies to the graduation classes of 2017 and onwards^b^
National	Industrial	Applies to the graduation classes up to and including the classes of 2016^a^. No equivalent orientations exist during the following years
	Media	
	Administration, commerce and logistics	Applies to the graduation classes of 2017 and onwards^b^. Equivalent orientations also existed in the previous years
	Art	As above
	Vehicle care and freight handling	As above
	Handicraft and production	As above
	Hotel, restaurant and bakery	As above
	Forest, land and animal	As above
	Property, installation and construction	Applies to the graduation classes of 2017 and onwards^b^. No equivalent orientations existed in the previous years
	Health, healthcare and nursing	As above
	Society, nature and language	As above
Specially designed	Local orientations. Goals not set by the government (National Agency for Education (Sweden), 2002).	Applies to the graduation classes up to and including the classes of 2016.^a^ Eleven different orientations were registered, also including ‘without any orientation’ and ‘other orientations’
Degree	Complete or incomplete	Applies to the graduation classes of 2001–2019^c^
Integrated studies	Information on whether at least half of the study time in USSID was spent in mainstream upper secondary school.	Applies to the graduation classes of 2020

Abbreviations: HURPID, Halmstad University Register on Pupils with Intellectual Disability; PIN, personal identity number.

^a^ Some individuals left these orientations later than 2016, probably due to study breaks or similar.

^b^ Some school units offered this orientation during a transition period, preceding the introduction of the new orientations in connection with the reform of USSID in 2013 (Ordinance on the curriculum for USSID, 2013; Swedish Education Act, 2010). As a result, some individuals left this orientation prior to 2017.

^c^ Starting with the graduating classes of 2017, the only reason for an incomplete degree is interrupted studies.

Swedish PINs enable HURPID to be linked with other PIN‐based registers. Table 2 provides an overview of the population‐based health and administrative registers and databases to which HURPID has been linked.

**TABLE 2:** Registers and databases that HURPID has been linked with.

Data provider	Register/database	Available information
National Board of Health and Welfare (Sweden)	National Register of Municipal Support and Service for Persons with Certain Functional Impairment (LSS)	Includes information regarding decided and implemented measures according to the Support and Service for Person with Certain Functional Impairments Act [LSS] (1993), from 1999 onwards, with some exceptions (National Board of Health and Welfare, 2022, October 20)
	National Patient Register (NPR)	Includes diagnoses reported by physicians in Swedish inpatient care since 1964 (comprehensive from 1987), specialised outpatient care since 2001, compulsory psychiatric care since 2010 and emergency services since 2016 (National Board of Health and Welfare, 2018, November 9)
	Swedish Medical Birth Register	Includes information on mothers and live‐born offspring from approximately 98% of all births in Sweden since 1973 (Cnattingius et al., 2023)
	Swedish Prescribed Drug Register	Includes information on prescribed drugs, profession and practice of the prescriber as well as PIN of the patient since 2005 (Wettermark et al., 2007)
	Swedish Cause of Death Register	Includes information on date and underlying cause of death of all deaths in Sweden since 1952 (Brooke et al., 2017)
Statistics Sweden	Longitudinal integration database for health insurance and labour market studies (LISA)	Includes information from the labour market, education, and social sector since 1990 on individuals, aged 16 or older, registered in Sweden (Statistics Sweden, 2019)
	Total Population Register (TPR)	Includes data on birth, place of residence, citizenship (including changes), civil status, family relationships, migration, confidential/protected identity, date of death etc. for Swedish inhabitants since 1961 (comprehensive from 1968). With some exceptions, emigrated individuals, staying abroad for more than 1 year, are de‐registered (Ludvigsson et al., 2016)
	Geography database (GDB)	Includes information on all properties (since 1983) and addresses (since 2000) in Sweden as of 1 Jan each year, also encompassing coordinates and geographical divisions (Statistics Sweden, 2023)
	Multi‐Generation Register	Includes parental information (PIN, date and country of birth, number of children etc.) on individuals, registered as residents in Sweden, who, with some exceptions, were born in 1932 or later (Ekbom, 2011)
Military Archives of Sweden; Swedish Defence Conscription and Assessment Agency	Swedish Military Conscription Register (SMCR)	Includes conscription information on physical and psychological health. Conscription was mandatory for all Swedish men, at about the age of 18, in 1969–2010. Data is available for 90% of these men, born in 1951–1987. Since 2017, conscription has been mandatory, regardless of legal gender, but the percentage called for standardised testing has been low (Ludvigsson et al., 2022)
Swedish National Council for Crime Prevention	National Crime Register	Includes information on prosecuted individuals since 1973. Details of administrative fines are not included in the register (unpublished data made to order)
Region Stockholm	The clinical database Pastill	Includes, among other details, information on diagnoses according to the DSM and/or the ICD from in‐ and outpatient child and adolescent psychiatric care within Stockholm County since 2001 (Lundh et al., 2013)
	Region Stockholm's clinical database on habilitation services	Includes data on utilisation of habilitation services (all ages) in region Stockholm, due to different types of disabilities (comprehensive since 1998)
Karolinska Institutet	Swedish Twin Registry	Includes nationwide information on twins, born in 1886 or later, identified and invited to participate based on data initially from Church book records and thereafter from national birth registrations (Zagai et al., 2019)

Abbreviations: DSM, diagnostic and statistical manual of mental disorders; HURPID, Halmstad University Register on Pupils with Intellectual Disability; ICD, International Statistical Classification of Diseases and Related Health Problems; PIN, personal identity number.

### Validation process

To validate HURPIDs potential as a nationwide register of adults with ID in Sweden, it was linked with the NPR (National Board of Health and Welfare (Sweden), 2018), the LSS (National Board of Health and Welfare (Sweden), 2022), the Swedish Military Conscription Register (SMCR) (Ludvigsson et al., 2022) (provided by the Military Archives of Sweden and the Swedish Defence Conscription and Assessment Agency), the Total Population Register (TPR) (provided by Statistics Sweden) (Ludvigsson et al., 2016), and the Geography database (GDB) (Statistics Sweden, 2023). The distribution of legal gender, educational programmes, and proportions of individuals included in HURPID out of all secondary education pupils were compared with corresponding variables from the NAEs (1994–) statistics. Associations between school programmes in HURPID and ID severity levels in the NPR were also investigated.

The current range of school programmes in USSID were introduced in 2013 (Ordinance on the curriculum for USSID, 2013) and consist of national, grade‐based programmes, and an individual, non‐grade‐based programme (Swedish Education Act, 2010; Upper secondary school ordinance, 2010). All national programmes include some aspect of theory (Swedish Education Act, 2010), which makes them more cognitively demanding than the individual programme, which is aimed at pupils assessed as not able to attend a national programme (Swedish Education Act, 2010). Before 2013, there were also specially designed programmes (NAE 2006a), which corresponded to the national ones in terms of educational level. There were also two different orientations within the individual programme; vocational training (offered grade‐based subjects) and general life skills training (offered non‐grade‐based subjects) (Ordinance concerning USSID, 1994).

The HURPID population was matched with 10 controls from the TPR for comparisons. The controls had to be of the same legal gender, born in the same calendar year, and registered as residents in the same municipality as those in HURPID at their expected graduation. Information about municipalities was obtained from the GDB. Duplicates among controls, likely due to narrow matching variables, were excluded and not replaced, which resulted in control case:ratio comparisons of 2:1 (one case), followed by 3:1 (three cases), 4:1 (16 cases), 5:1 (84 cases), 6:1 (309 cases), 7:1 (925 cases), 8:1 (2976 cases), 9:1 (7815 cases) and 10:1 (14,701 cases).

The occurrence of the following diagnoses in the NPR between 1987 and 2020, coded according to the Swedish versions of the International Statistical Classification of Diseases and Related Health Problems (ICD), was examined in the HURPID population: ID (ICD‐9 codes 317–319 and ICD‐10 codes F70–F73 and F78–F79), overactive disorder associated with mental retardation and stereotyped movements (ICD‐10 code F84.4), Rett syndrome (ICD‐10 code F84.2), ASD (ICD‐9 code 299A and ICD‐10 codes F84.0, F84.1, F84.3, F84.5, F84.8 and F84.9), chromosomal abnormalities (ICD‐9 code 758 and ICD‐10 codes Q90–99 (not separable)), and traumatic brain injury (ICD‐9 codes 800–804, 850–854, and ICD‐10 codes S02.0, S02.1, S02.7, S02.8, S02.9, S04.0, S06 and S07.1 (World Health Organisation [WHO], 1986, 2024) (not separable). The selected diagnoses were based on their associations with the USSID target groups and their availability in an existing linkage. Since the diagnosis traumatic brain injury also includes milder brain injuries, such as concussions (codes 850 and S06) (WHO, 1986, 2024), which are unlikely to result in cognitive impairments, this variable was solely used to examine the characteristics of the HURPID population. Categorisations into mild (codes 317 and F70) and more severe levels of ID (codes 318 and F71–F73) (WHO, 1986, 2024) were based on the most recent diagnosis. Those with both a mild and a more severe or unspecified level of ID registered at the latest registration date were treated as missing.

The reason for the latest approved application according to the Support and Service for Person with Certain Functional Impairments Act (LSS, 1993) in 1999–2003 and 2007–2020 were examined in the LSS (National Board of Health and Welfare (Sweden), 2022). Priority group 1 comprises individuals with ID/ASD (not separable). Priority group 2 comprises individuals with acquired brain injury, caused by external impact (traumatic brain injury) or by disease, that resulted in significant and permanent intellectual impairment (LSS, 1993).

For individuals called for standardised testing at the military conscription process in Sweden up to and including the year 2020, the recorded stanine scores on the intelligence test in the SMCR (Ludvigsson et al., 2022) were examined. Intellectual functioning corresponding to two standard deviations or more below the average, that is results equivalent to stanine 1, is one of three criteria that must be met to diagnose ID (APA, 2022; WHO, 1992).

The chi‐square test for goodness of fit was used for comparisons between the original version of HURPID in 2022 and the expected values according to the NAE (1994–). The chi‐square test for independence was used for comparisons between groups based on a linkage where 80 individuals were excluded from HURPID for various reasons. This test was also used for investigation of associations between school programmes and ID severity levels.

## RESULTS

**FIGURE 1:**
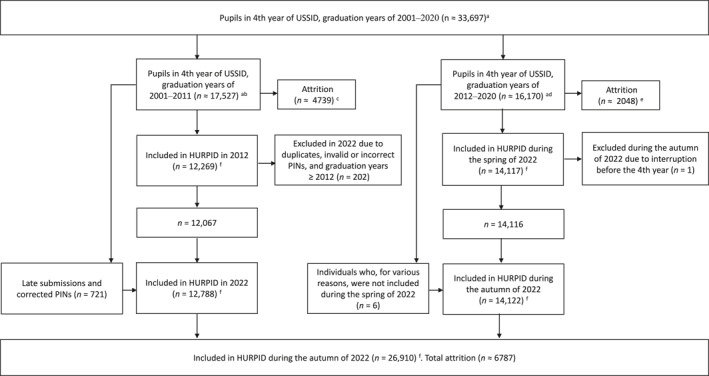
Flow chart of the Halmstad University Register on Pupils with Intellectual Disability (HURPID). PIN, personal identity number; USSID, Upper Secondary School for pupils with Intellectual Disability. ^a^Since this approximation is based on non‐individual‐specific data, reported by the school organisers about 8 months prior to graduation (National Agency for Education (Sweden), 1994–), it is not possible to determine the accuracy of this statistics. ^b^Some potential underreporting was identified (*n* = 9). ^c^Attrition due to invalid PINs (*n* = 69), that no data containing at least a valid PIN were received from the organiser/organisers in the municipality (*n* ≈ 147), late submissions that not yet have been included (*n* > 197), or unknown reason. ^d^Some potential overreporting, detected during the establishment of HURPID (probable duplicates in the form of individuals who seemed to have attended the fourth year more than once), or acknowledged by the school organisers, was identified (*n* = 18). ^e^Attrition, partly due to invalid PINs (*n* = 44). ^f^Some individuals who discontinued their studies before entering year four might have been included.

HURPID contains information on 26,910 individuals (12,788 individuals from the graduation classes of 2001–2011 and 14,122 from the classes of 2012–2020). The school organisers' reported *n* = 17,527 in the graduation classes of 2001–2011 and *n* = 16,170 in 2012–2020. Overall, the NAE (1994–) estimated that 33,697 individuals attended the graduation classes of 2001–2020 (completeness of about 80% in HURPID). Figure 1 shows the participant flow and explanations for missing data.

**FIGURE 2:**
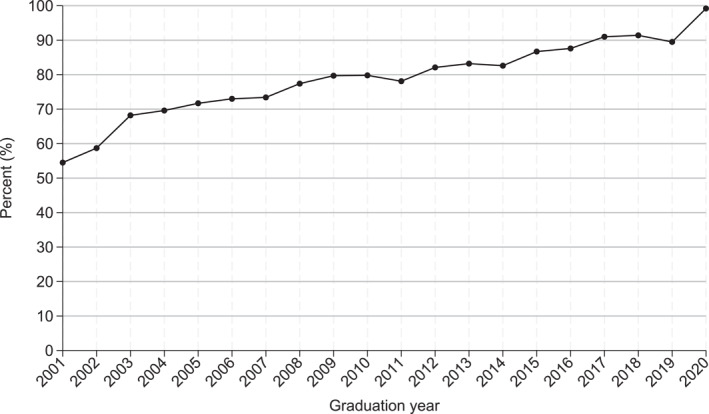
Variation in completeness in Halmstad University Register on Pupils with Intellectual Disability (HURPID) across the graduation years. Completeness was estimated as the number of individuals in HURPID (*n* = 26,907), divided by the number of individuals in the fourth year of Upper Secondary School for pupils with Intellectual Disability, as reported by the school organisers’ about eight months before graduation. Up to and including the graduation year of 2019, this data comprised non‐individual‐specific statistics (National Agency for Education (Sweden), 1994–).

Figure 2 shows the variation in completeness in HURPID across the graduation years 2001–2020. The completeness ranged between 54.5% and 99.2%. Although individual‐specific data were obtained from the NAE for 2020, completeness did not reach 100% due to invalid PINs (*n* = 12).

**FIGURE 3:**
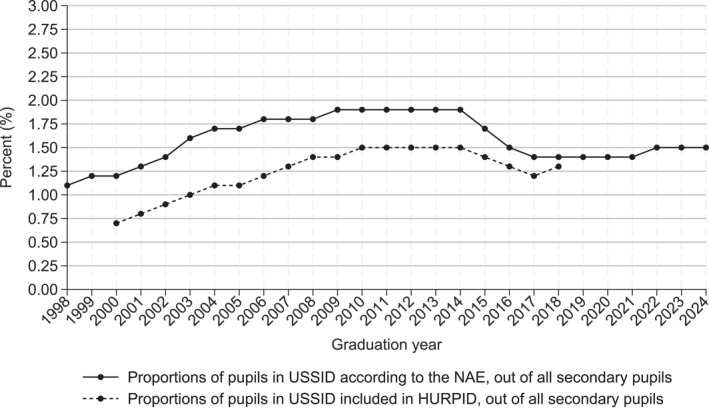
In 1968, all children and young people in Sweden were given the statutory right to education, regardless of their cognitive functioning (Law regarding care for certain people with intellectual disabilities, 1967). The solid line shows the proportions of pupils in Upper Secondary School for pupils with Intellectual Disability (USSID) out of all secondary education pupils in Sweden, distributed by graduation year, according to statistics from the National Agency for Education (Sweden) [NAE] (1994–). The dashed line shows the proportions of pupils in USSID included in HURPID out of all secondary education pupils in Sweden (1994–), distributed by graduation year. In some years, the Compulsory School for Pupils with Intellectual Disabilities and USSID have covered one additional year each compared to mainstream options (Swedish Education Act, 1985, 2010). Thus, to give an as fair picture of the proportions as possible, the number of pupils in the second and third years of USSID was compared to all secondary education pupils in year two and three (NAE, 1994–). Such calculations cannot be made earlier than 1998, as prior to that, there were also two‐year options available in the mainstream upper secondary school (Government of Sweden, 1990). Please note that information regarding schooling in the years two and three is not available in HURPID. Based on the recorded graduation years of the individuals in HURPID, a calculation was therefore made to estimate when they were expected to have attended year two and three. The proportion of individuals in graduation year 2018, for example, was calculated as the number of individuals in HURPID who, in 2018, were expected to have attended years two and three in USSID (i.e., those who are registered under graduation years 2019 and 2020 in HURPID), divided by all secondary education pupils in years two and three (NAE, 1994–). Please also note that not all pupils in USSID years two and three continue their studies in year four. For instance, 33,697 individuals attended year four during the graduation years of 2001–2020, according to the NAE (1994–). These individuals are expected to have been in year three between 2000 and 2019 and in year two between 1999 and 2018. However, according to the NAE (1994–), the number of pupils in year three was 35,285 between 2000 and 2019, and the number of pupils in year two was 37,068 between 1999 and 2018. This corresponds to a decrease of 3371 pupils (9.1%). In addition to actual attrition, this helps explain the lower prevalence figures represented by the dashed line compared to the solid line.

Figure 3 illustrates the variations in the proportions of pupils in USSID, based on statistics from the NAE (1994–), alongside the proportions of pupils included in HURPID, as shares of all secondary education pupils in Sweden, across different years (NAE (Sweden), 1994–). The proportions of pupils included in HURPID ranged between 0.7% and 1.5%. The proportions of pupils in USSID (NAE (Sweden), 1994–), ranged between 1.2% and 1.9% during the same period.

Characteristics of the HURPID population, the corresponding population according to the NAE (1994–) and the general population are presented in Table 3.

**TABLE 3:** Characteristics of the HURPID population (graduation classes of 2001–2020), compared to the corresponding statistics of the NAE and to controls.

Variables	HURPID	NAE	Controls
	*n* = 26,910^a^	*n* = 33,697^b^	
	*n* = 26,830^c^		*n* = 249,977^d^
Legal gender	*n* = 26,910	*n* = 33,697	*n* = 249,977
Women	41.2%	41.1%^e^	41.5%
Men	58.8%	58.9%^e^	58.5%
	*p1* = 0.953		
Programmes	*n* = 25,149	*n* = 33,697	
Grade‐based^f^	80.0%	77.6%	N/a
Non‐grade‐based^g^	20.0%	22.4%	N/a
	*p1* < 0.001		
Municipalities	*n* ^h^ = 191–192^i^	*n* ^h^ = 207	N/a
Diagnoses^j^	*n* = 26,830		*n* = 249,977
ID	56.1%	N/a	0.6%
	*p2* < 0.001		
ASD	22.0%	N/a	2.3%
	*p2* < 0.001		
ASD (not ID)	5.3%	N/a	2.1%
	*p2* < 0.001		
Chromosomal abnormalities	10.7%	N/a	0.1%
	*p2* < 0.001		
Traumatic brain injury	9.6%	N/a	9.2%
	*p2* = 0.063		
LSS Support	*n* = 26,830		*n* = 249,977
Priority group 1^k^	77.1%	N/a	1.4%
Priority group 2^l^	0.07%	N/a	0.01%
Priority group 3^m^	0.4%	N/a	0.1%
	*p2* < 0.001		
Intellectual ability^n^	*n =* 464		*n =* 48,156
Not completed	*	N/a	*
1	52.6%	N/a	2.9%
2–5	47.2%	N/a	57.3%
6–9	*	N/a	39.8%
	*p2* < 0.001		
Associated with USSID target groups in ≥ 1 register	*n* = 26,830		*n* = 249,977
	84.6%	N/a	3.6%
	*p2* < 0.001		

*Note*: *p1* = *p*‐value resulting from comparisons with the expected value according to the NAE (1994–), based on the chi‐square test for goodness of fit; *p2 = p‐*value resulting from comparisons with controls, based on the chi‐square test for independence; * = *n* < 5.

Abbreviations: ASD, autism spectrum disorder; HURPID, Halmstad University Register on Pupils with Intellectual Disability; ID, intellectual disability; LSS, the Support and Service for Person with Certain Functional Impairments Act (LSS, 1993); NAE, National Agency for Education.

^a^ Included in HURPID since the autumn of 2022.

^b^ As reported by the school organisers (NAE, 1994–).

^c^ Available for comparisons with the NPR och with the controls.

^d^ Matched by legal gender, year of birth, and registered municipality of residence at the time of the cases' graduation. The number of controls per case (*n* = 26,830) varied between 2 and 10.

^e^ Information on legal gender was obtained from Statistics Sweden (unpublished data made to order) for the graduation classes of 2001–2011, and from the NAE (1994–) for the graduation classes of 2012–2020.

^f^ National programmes, specially designed programmes and individual programme with an orientation in vocational training.

^g^ Individual programme with an orientation in general life skills training or individual programme without any orientation.

^h^ Number of municipalities where the schools were located. Sweden consists of 290 municipalities.

^i^ The municipalities were situated in the whole of Sweden and were of different sizes. The school organiser in one of the municipalities had not reported having any pupils in the fourth year of USSID (unpublished data made to order). The number is uncertain because it was not possible to ascertain whether one upper secondary school association had submitted documents for both of its school units, which were in different municipalities.

^j^ Recorded in the National Patient Register between 1987 and 2020.

^k^ Approved for support due to ID or ASD.

^l^ Approved for support due to acquired brain injury.

^m^ Approved for support due to other reason than ID, ASD or an acquired brain injury.

^n^ Recorded on the intelligence test in the Swedish Military Conscription Register up until 2020.

In addition to being assessed as belonging to the previous or current USSID target groups, 84.6% of the individuals in HURPID (*n* = 26,830) were identified in at least one other register associated with these target groups. When excluding individuals without ID in the NPR who, at the latest assessment, were approved for support according to the LSS (1993) due to priority group 2 (acquired brain injury), or had a registered ASD diagnosis in the NPR, the proportion was 84.0%.

**FIGURE 4:**
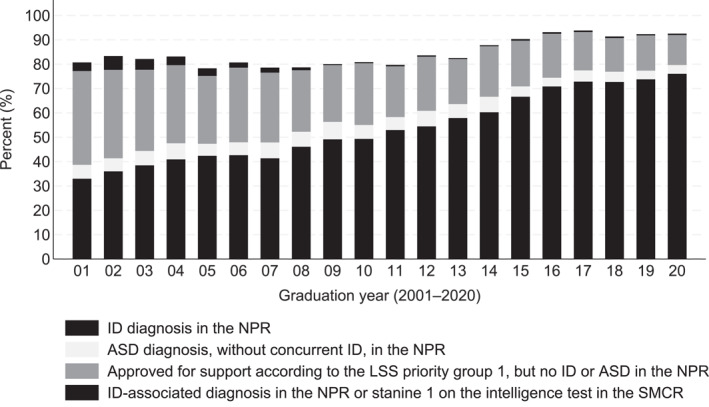
Proportions of individuals in the Halmstad University Register on Pupils with Intellectual Disability identified in at least one additional register associated with the target group of Upper Secondary School for pupils with Intellectual Disability, distributed by graduation year (*n* = 26,828). Priority group 1 comprises individuals who, at the latest assessment, was approved for support according to the Support and Service for Person with Certain Functional Impairments Act (LSS, 1993) due to ID or ASD. ASD, autism spectrum disorder; ID, intellectual disability; NPR, National Patient Register; SMCR, Swedish Military Conscription Register.

Figure 4 shows how the proportions of individuals in HURPID (*n* = 26,828) who were identified in at least one additional register associated with the previous or current USSID target groups were distributed across the graduation years. The proportions ranged from 77.5% in 2005 to 93.6% in 2017. The number of individuals without a registered ID‐diagnosis in the NPR, that, at the latest assessment, was approved for support according to the LSS (1993) due to priority group 2 (acquired brain injury) was too small to be displayed in Figure 4 (*n* = 11 (0.04%)). About half of these had a registered traumatic brain injury in the NPR (*n* = 5).

For 11,679 individuals in HURPID (total *n* = 26,830) there were consistent records both regarding educational programme (including information on potential orientation within the individual programme) and ID diagnosis (including severity level). Table 4 shows the associations between educational programmes and ID severity levels, based on two different programme categorisations.

**TABLE 4:** Associations between educational programmes in USSID and ID severity levels based on two different programme categorisations (*n* = 11,679).

Programme categories	Total, *n*	ID severity levels	
		Mild, *n* (%)	Moderate or more severe, *n* (%)
NP/SM^a^	6716	6330 (94.2)	386 (5.8)
Individual^b^	4963	2169 (43.7)	2794 (56.3)
Grade‐based^c^	8981	7807 (86.9)	1174 (13.1)
Ungraded^d^	2698	692 (25.6)	2006 (74.3)

*Note*: There was a statistically significant association between both types of educational programme categorisations registered in the Halmstad University Register on Pupils with Intellectual Disability (HURPID) and intellectual disability (ID) severity level registered in the National Patient Register (NPR) (χ^2^(1) = 3700.00, *p* < 0.001 for the NP/SM and individual programmes categorisation, and χ^2^(1) = 3900.00, *p* < 0.001 for the grade‐based and ungraded programmes categorisation).

Abbreviation: USSID, Upper Secondary School for pupils with ID.

^a^ The national (NP) and the specially designed (SM) programmes correspond to each other in terms of educational level (Ordinance concerning USSID, 1994).

^b^ Aimed at pupils assessed as not having the ability to attend another programme (Swedish Education Act, 2010).

^c^ Include national and specially designed programmes, as well as an orientation within the individual programme (vocational training).

^d^ Include the individual programme without any orientation, and the general life skills training orientation.

There was a statistically significant association between the two educational programme categorisations and ID severity level (χ^2^(1) = 3700.00, *p* < 0.001 for the national/specially designed and individual programmes categorisation, and χ^2^(1) = 3900.00, *p* < 0.001 for the grade‐based and ungraded programmes categorisation). The largest proportion of individuals with mild ID was found among those who had attended a national or a specially designed programme (94.2%). The largest proportion of individuals with a moderate or a more severe level of ID was found among those who had attended a non‐grade‐based programme (74.3%).

### Knowledge contributions from projects involving HURPID

New insights into occupational patterns after attending USSID have emerged, primarily based on the initial data collection period. A cross‐sectional study in 2011 found that nearly half (46.9%) of the individuals participated in daily activities under the LSS (1993), while the remainder were employed (22.4%), studying (6.6%), or had no known occupation (24.1%) (Arvidsson et al., 2015). Occupational group affiliations were associated with legal gender (Arvidsson, Widén, et al., 2016), municipality of residence (Arvidsson, Staland‐Nyman, et al., 2016), parents' educational level, and country of birth (Arvidsson et al., 2020). Educational programme (Luthra et al., 2018) and family situation (Luthra, 2019) were associated with the group without a known occupation. Follow‐up on employment showed a gradual increase of nearly 10% between 2011 and 2020 (Tideman et al., 2024). Another follow‐up study, incorporating data from both data collection periods, found that the proportion participating in daily activities after leaving USSID remained stable (Luthra et al., 2024).

In a study on psychiatric comorbidity, it was found that individuals with ID were exposed to a high risk of co‐occurring psychiatric disorders (Ivarsson et al., submitted for publication). Excess premature mortality was also found within this group (Hirvikoski et al., 2021), as was an increased risk of ID among relatives of individuals with ID (Lichtenstein et al., 2022). In addition, it was found that individuals with mild ID were at increased risk of substance use‐related problems (Påhlsson‐Notini et al., 2024), and that ID was associated with elevated risks of violent and sexual crimes and assault victimisations (Latvala et al., 2023).

Using register analysis results, in‐depth qualitative studies have been conducted on the groups in employment, daily activities, and without known occupation. Five facilitators supporting employment sustainability were identified: having tried various jobs, enjoying being at work, balanced expectations and adaptations, mutual engagement and flexibility, and wage subsidies (Taubner et al., 2023). Meaningful social relationships contributed to a sense of belonging, influenced by factors related to having an ID, young adulthood, and occupational status among individuals without a known occupation (Luthra, 2024).

## DISCUSSION

### Key results

HURPID offers unique opportunities to gain essential knowledge about one of society's most marginalised groups. This article aimed to describe HURPIDs construction, structure and content (graduation classes of 2001–2020), validate its potential as a nationwide register of adults with ID in Sweden, and provide examples of the knowledge contributed by various projects involving HURPID.

Currently, HURPID includes 26,910 individuals (11,078 women and 15,832 men, born in 1973–2003). The exact number in the target population is unknown, as Swedish authorities were not permitted to collect individual‐specific information on USSID attendees until autumn 2019 (Föreskrifter om ändring i Skolverkets föreskrifter (SKOLFS 2011:142) om uppgiftsinsamling från huvudmännen inom skolväsendet m.m., 2019). However, the NAE (1994–) estimated that the expected number of individuals in the USSID graduation classes of 2001–2020 was 33,697. This represents 80% completeness in HURPID.

Several reasons for the attrition have been identified, such as invalid PINs, school organisers that did not submit any documentation for deceased individuals or individuals with protected personal data, and school organisers who failed to identify some former pupils. Some school organisers included only individuals registered in their own municipality or excluded those who had attended non‐graded programmes. Some independent schools chose not to participate or were unreachable, and some data has yet to be included. Potential duplicates were also identified, as some individuals appeared to have attended the fourth year more than once. Moreover, individuals who seemed to have been enroled in USSID but only had obtained grades from mainstream upper secondary school courses were excluded.

The reasons mentioned do not fully account for the difference between the number of individuals in HURPID and the estimated figure from the NAE (1994–). In addition to the previously noted uncertainty in this estimation, other possible explanations exist. One factor concerns the time elapsed since the requested documents were issued. Both data collection periods demonstrated the lowest inclusion rates from the earliest graduation cohorts, compared to the statistics from the NAE (1994–). Another possible factor relates to the fact that the municipality responsible for USSID admissions is often not the school organiser or issuer of school leaving documents. This raises uncertainty about whether all those concerned received the request. Furthermore, follow‐up questions to school organisers not contacted due to time constraints could have provided additional data. Estimating the extent to which these factors account for the difference remains challenging.

The distribution of women and men in HURPID matched the estimate of the NAE (1994–). The proportion who had attended a grade‐based programme was significantly higher in HURPID. The proportion who had attended a non‐grade‐based programme was significantly lower. An ID diagnosis was recorded in the NPR for 15,055 individuals (56.1%) in HURPID. Among these, severity level was specified for 12,355 individuals (73.5% mild, 26.5% more severe level). It is likely that the proportion of individuals with mild ID in HURPID would have been higher if diagnoses from healthcare providers not reporting to the NPR were also included. When using national and specially designed programmes as a proxy for mild ID, 94.2% of the individuals in HURPID (*n* = 11,679) with this diagnosis was identified in the NPR. The largest proportion of individuals with a more severe level of ID was found among those who had attended a non‐grade‐based programme (74.3%).

The schools represented in HURPID were geographically spread across Sweden, in municipalities of various sizes. This reflects the population‐based nature of the register.

In addition to being assessed as belonging to the USSID target group, 84.6% of the individuals in HURPID were identified within at least one of the registers available for the study, associated with the previous or current USSID target group. The number of remaining individuals diagnosed with ID in health care settings that do not report to the NPR is unknown.

There are several possible reasons why the proportions of pupils in USSID, according to the NAE (1994–), and the proportions of individuals included in HURPID, out of all secondary education pupils in Sweden, both fluctuate and, in certain years, exceed the expected global prevalence of ID (approximately 1%). Prevalence rates vary by age (APA, 2022; Maulik et al., 2011; McKenzie et al., 2016). The highest rates are reported for children and adolescents globally (APA, 2022; Maulik et al., 2011) (almost 2%) (Maulik et al., 2011). During the mid‐1990s, the total proportion of pupils in the Compulsory School for Pupils with Intellectual Disabilities in Sweden increased from approximately 0.8% to 1.4%. Some municipalities assessed as many as 3.6% of their registered pupils to be eligible for this school form. This also led to an increase in the proportion of pupils in USSID. Factors such as improved statistics, financial incentives, a new grading system, changes in teaching and organisation, a rise in ASD diagnoses, a new school organiser and deficient investigative procedures are assumed to explain the increase (NAE, 2006b). According to this study, however, ASD diagnoses do not seem to account for much of this increase.

### Limitations

One limitation concerns the inability to ascertain whether the significantly lower proportion of individuals having attended non‐grade‐based programmes in HURPID, compared to the approximation of the NAE, results from inaccuracies in the approximation, systematic attrition, or registration errors due to incompatible combinations of course codes and programmes on the documents for example. The proportion of individuals in HURPID with a specified ID severity level do not indicate that individuals with more severe levels of ID would be missing from HURPID though.

Another limitation concerns the diagnoses of the individuals included in HURPID. HURPID is based on information that confirms school attendance within USSID, but the reason for enrolment (ID, acquired brain injury or ASD) is not known. Over half of the HURPID individuals' ID‐diagnoses (although not their current validity) could be confirmed when comparing with the NPR. An additional near third was linked to other diagnoses or variables associated with the target group of USSID. It is possible that including diagnoses registered prior to 1987, as well as more precisely defined acquired brain injuries due to various causes, might have identified a few more cases. However, most individuals who were not identified via the NPR are likely to have been diagnosed in healthcare settings that do not report to that register. This was expected and confirms the need for a register like HURPID.

## CONCLUSION

### Interpretation

To our knowledge, HURPID demonstrates the highest completeness of individuals with ID of working age in Sweden and constitutes one of the most extensive and comprehensive, nationwide registers of individuals with ID in the world. This makes HURPID unique both in a Swedish and in an international context. Linking the register with any other register based on the Swedish PIN enables knowledge advancement across many different domains through registry research. Although the practical applicability of HURPID is limited to individuals with Swedish PINs, findings from such research can, in turn, lead to in‐depth qualitative studies and implications for practice both nationally and internationally. Moreover, studies including HURPID can, for instance, serve as a reference in international comparisons and contribute to assessing the robustness of findings from more fragmented cohorts in other countries.

The validation showed that most individuals in HURPID (84.6%, total *n* = 26,830) could be linked to variables associated with the current or previous target groups of USSID (ID, acquired brain injury or ASD). The proportion of individuals with ASD, without concurrent ID, in the NPR, or acquired brain injury (priority group 2) in the LSS (National Board of Health and Welfare, 2022), was 5.3%. Only a very small proportion of the individuals in HURPID are expected to have attended USSID due to an acquired brain injury. This argues for the continued use of HURPID as a register of individuals with ID.

### Generalisability

HURPID is considered a nationwide, comprehensive register of the cohorts covered. The register is regarded as well representative in terms of legal gender, compared to the approximation of Statistic Sweden (unpublished data made to order) and the NAE (1994–). The variables of national and specially designed programmes can be used in combination as a proxy to identify individuals with mild ID. It is not, with the same degree of certainty, possible to identify individuals with more severe levels of ID based on school programme.

It cannot be ruled out that there may have been a systematic attrition among individuals who attended non‐grade‐based programmes. This should be taken into consideration when generalising results based on HURPID. No other risk of systematic attrition has been identified.

## AUTHOR CONTRIBUTIONS

**Eva Jönsson**: Data curation; formal analysis; investigation; methodology; writing—original draft. **Carin Staland‐Nyman**: Investigation; methodology; writing—review and editing. **Paul Lichtenstein**: Methodology; project administration; writing—review and editing. **Magnus Tideman**: Conceptualization; funding acquisition; investigation; methodology; project administration; supervision; writing—review and editing.

## CONFLICT OF INTEREST STATEMENT

The authors declare no conflicts of interest.

## ETHICAL CONSIDERATIONS

Ethics approvals for the first round of data collection and for linking HURPID with other registers were received from the Regional Ethics Review Boards in Lund and Stockholm, Sweden (Record numbers 2011/326, 2011/782, 2013/862‐31/5 and 2016/1214‐32). Ethics approval for the supplementation of orientations for the national and specially designed programmes was obtained from the Regional Ethics Review Board in Lund (Record number 2014/691). Ethics approvals for the second round of data collection and for linking the expanded register with other registers were received from the Swedish Ethics Review Authority (Record numbers 2020‐01838, 2020–03932, 2023‐01390‐02 and 2020–06540). HURPID was established in accordance with the principles described in the Declaration of Helsinki. Information does not need to be provided to individuals who are listed in registers if it involves a disproportionate amount of work, according to both previous (the Personal Data Act) and current legislation (the General Data Protection Regulation). This was justified in the ethics applications and approved (Record numbers 2011/326 and 2020‐01838).

## Data Availability

No decision has yet been made regarding future data access, but collaborations are encouraged. New linking and ethics approval will be required to access the latest version of the register. For further information, please contact Professor Magnus Tideman, E‐mail: magnus.tideman@hh.se.
